# Drastic antitumor response following administration of afatinib immediately after atezolizumab in a patient with epidermal growth factor receptor tyrosine kinase inhibitor‐resistant lung cancer

**DOI:** 10.1111/1759-7714.14047

**Published:** 2021-06-06

**Authors:** Toshiyuki Sumi, Hisashi Nakata, Hirofumi Chiba

**Affiliations:** ^1^ Department of Pulmonary Medicine Hakodate Goryoukaku Hospital Hakodate Japan; ^2^ Department of Respiratory Medicine and Allergology Sapporo Medical University School of Medicine Sapporo Japan

**Keywords:** afatinib, atezolizumab, rechallenge

**FIGURE 1:**
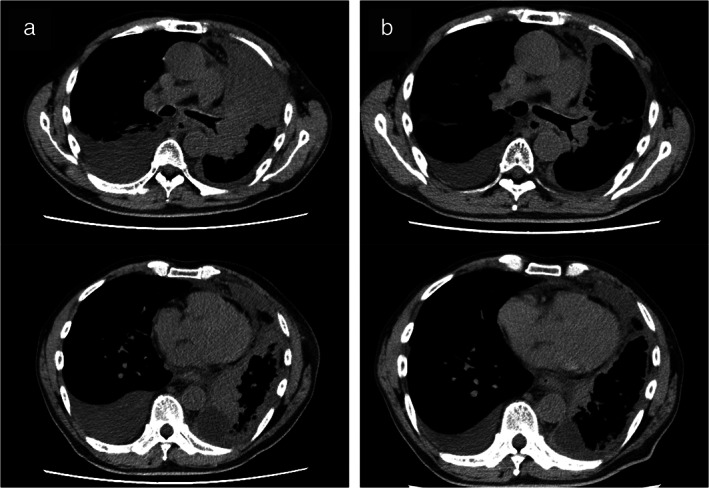
Chest computed tomography images before and after afatinib rechallenge therapy, and histological findings of a rebiopsy sample before afatinib rechallenge treatment. (a) Left hilar mass, left pleural metastasis, and right malignant pleural effusion were observed prior to the afatinib rechallenge. (b) Ten days after initiating afatinib rechallenge, reductions in the left hilar mass and left pleural metastasis were noted

A 61‐year‐old Asian man with a smoking history underwent chemoradiotherapy for stage IIIB (cT4N2M0) left upper lobe pulmonary adenocarcinoma harboring an exon 19 deletion in the epidermal growth factor receptor (*EGFR*) gene. In December 2013, he received carboplatin (area under the curve, 2), paclitaxel (40 mg/m^2^) on days 1, 8, and 15 for two 3‐week‐cycles, and radiotherapy (60 Gy distributed over 30 fractions, 5 days per week) as first‐line therapy. He subsequently presented with primary tumor regrowth in July 2015. Treatment with cisplatin, pemetrexed, and bevacizumab was initiated; however, after 13 months, right pulmonary metastasis recurred. He responded well upon being switched to afatinib (40 mg/day) for approximately four years before experiencing multiple relapses. The results of pleural fluid biopsies showed an exon 19 deletion but no T790M mutation. Therefore, in November 2020, afatinib was discontinued and the immune checkpoint inhibitor (ICI) atezolizumab (1200 mg/bodyweight, every 3 weeks) was prescribed. Two months later, chest computed tomography (CT) revealed that the primary tumor had grown and formed a hilar mass (Figure 1(a)). In January 2021, samples from the left upper lobe were biopsied; there was an exon 19 deletion, no T790M nutation, and PD‐L1 TPS 80%. A fifth strategy was initiated— rechallenge administration of afatinib (40 mg/day) immediately after atezolizumab. The hilar mass markedly diminished 10 days after commencing afatinib (Figure 1(b)). Response has been sustained for more than 12 weeks.

Recently, researchers have demonstrated that EGFR inhibition improves responsiveness to programmed cell death 1 (PD‐1) blockade in *EGFR*‐mutated non‐small cell lung cancer (NSCLC).^1^ EGFR signaling is important in driving high regulatory T cell (Treg) infiltration, despite low CD8^+^ effector T cell infiltration in *EGFR*‐mutated lung adenocarcinomas, via CCL22 upregulation through JNK/cJun and CXCL10 downregulation mediated by IRF1. EGFR–TKIs reduce the high levels of Treg infiltration associated with *EGFR* mutation‐positive NSCLC. Animal studies indicated a greater antitumor effect with combination EGFR–TKI/anti‐PD‐1 antibody therapy than with either therapy alone. Further, Tregs express EGFR in inflamed tumor microenvironments, and the mast cell‐derived epidermal growth factor‐like amphiregulin enhances Treg immunosuppressive effects, a phenomenon inhibited by EGFR–TKIs.^2^

**FIGURE 2:**
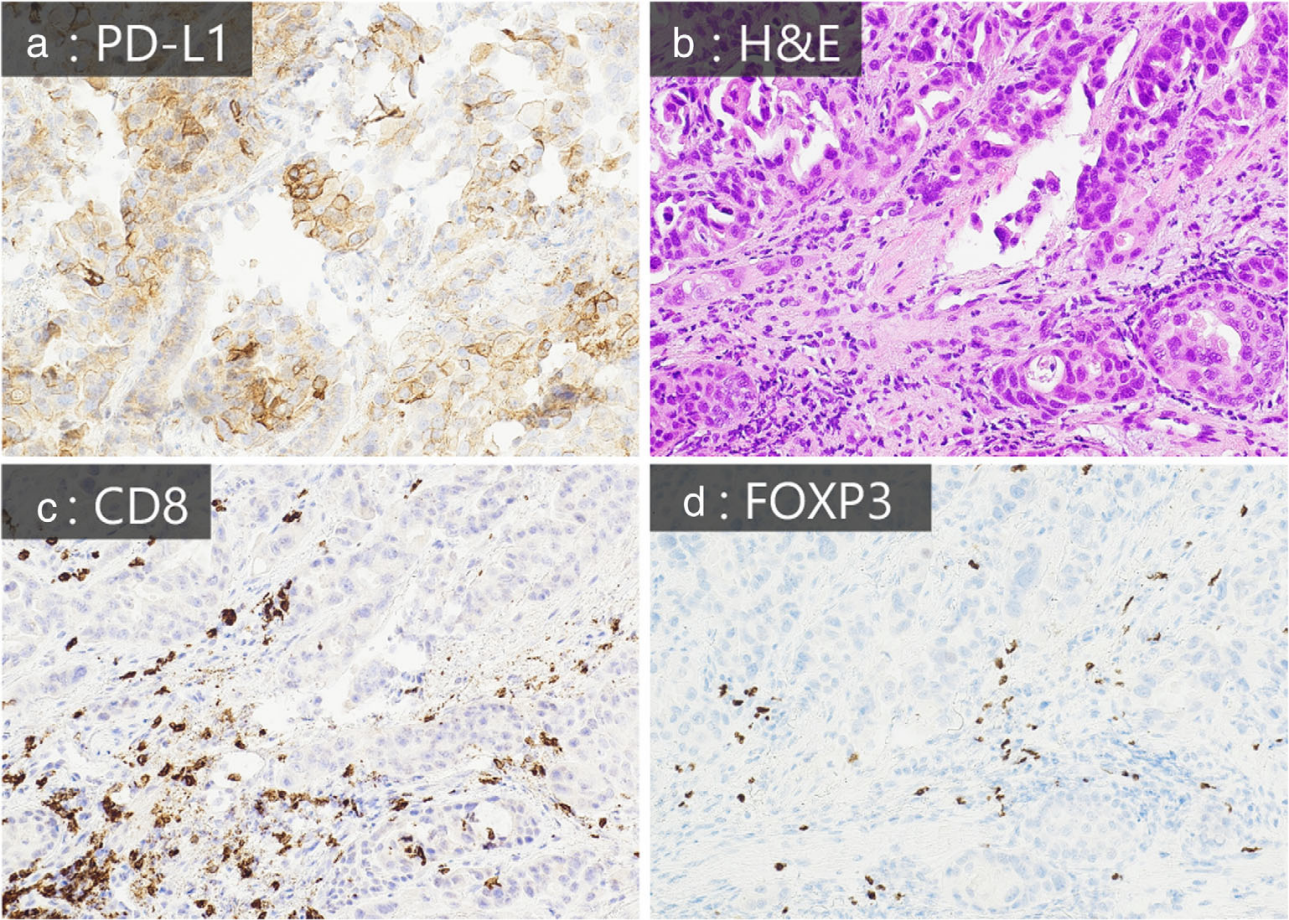
Histological findings of rebiopsy sample before afatinib rechallenge treatment. (a) Tumor cells highly expressing PD‐L1. (b) Lymphocytic infiltration in the tumor stroma. Hematoxylin–eosin staining, ×20. (c, d) Lymphocytes infiltrating the stroma include a mixture of CD8‐positive (c) and FOXP3‐positive cells (d)

Immunohistochemical staining of the rebiopsied tissue before afatinib treatment showed that tumor cells expressed high levels of PDL1, and CD8‐positive effector T cells infiltrated the tumor stroma but did not respond to anti‐PD‐L1 antibody. This might be because of simultaneous FOXP3‐positive T cell (Treg) infiltration (Figure 2(a)–(d)). Thus, although afatinib and atezolizumab were administered sequentially to our patient, EGFR inhibition by afatinib might have improved responsiveness to PD‐1 blockade induced by atezolizumab in EGFR–TKI‐resistant tumors. The safety of sequential anticancer drug administration must be investigated. Uchida et al.^3^ reported osimertinib‐induced interstitial lung disease (ILD) in three of 12 patients who received this third‐generation EGFR–TKI immediately after anti‐PD‐1 antibodies; however, EGFR–TKI‐induced ILD was not observed in five patients treated with first‐ or second‐generation EGFR–TKIs. Another study reported that in 13 patients treated with first‐ or second‐generation EGFR–TKIs immediately after anti‐PD‐1 antibody failure, the response rate was 46.1%, with no grade 3/4 adverse events or ILD.^4^ Therefore, first‐ and second‐generation EGFR–TKIs might be relatively safe, even if administered immediately after ICIs. In summary, rechallenge with a first‐ or second‐generation EGFR–TKI immediately after ICI therapy might be a tolerable and effective option for patients with EGFR–TKI resistance.
